# Exploring the Possible Use of AI Chatbots in Public Health Education: Feasibility Study

**DOI:** 10.2196/51421

**Published:** 2023-11-01

**Authors:** Francesco Baglivo, Luigi De Angelis, Virginia Casigliani, Guglielmo Arzilli, Gaetano Pierpaolo Privitera, Caterina Rizzo

**Affiliations:** 1 Department of Translational Research and New Technologies in Medicine and Surgery University of Pisa Pisa (PI) Italy; 2 Training Office National Institute of Health Rome Italy

**Keywords:** artificial intelligence, chatbots, medical education, vaccination, public health, medical students, large language model, generative AI, ChatGPT, Google Bard, AI chatbot, health education, public health, health care, medical training, educational support tool, chatbot model

## Abstract

**Background:**

Artificial intelligence (AI) is a rapidly developing field with the potential to transform various aspects of health care and public health, including medical training. During the “Hygiene and Public Health” course for fifth-year medical students, a practical training session was conducted on vaccination using AI chatbots as an educational supportive tool. Before receiving specific training on vaccination, the students were given a web-based test extracted from the Italian National Medical Residency Test. After completing the test, a critical correction of each question was performed assisted by AI chatbots.

**Objective:**

The main aim of this study was to identify whether AI chatbots can be considered educational support tools for training in public health. The secondary objective was to assess the performance of different AI chatbots on complex multiple-choice medical questions in the Italian language.

**Methods:**

A test composed of 15 multiple-choice questions on vaccination was extracted from the Italian National Medical Residency Test using targeted keywords and administered to medical students via Google Forms and to different AI chatbot models (Bing Chat, ChatGPT, Chatsonic, Google Bard, and YouChat). The correction of the test was conducted in the classroom, focusing on the critical evaluation of the explanations provided by the chatbot. A Mann-Whitney U test was conducted to compare the performances of medical students and AI chatbots. Student feedback was collected anonymously at the end of the training experience.

**Results:**

In total, 36 medical students and 5 AI chatbot models completed the test. The students achieved an average score of 8.22 (SD 2.65) out of 15, while the AI chatbots scored an average of 12.22 (SD 2.77). The results indicated a statistically significant difference in performance between the 2 groups (U=49.5, *P*<.001), with a large effect size (r=0.69). When divided by question type (direct, scenario-based, and negative), significant differences were observed in direct (*P*<.001) and scenario-based (*P*<.001) questions, but not in negative questions (*P*=.48). The students reported a high level of satisfaction (7.9/10) with the educational experience, expressing a strong desire to repeat the experience (7.6/10).

**Conclusions:**

This study demonstrated the efficacy of AI chatbots in answering complex medical questions related to vaccination and providing valuable educational support. Their performance significantly surpassed that of medical students in direct and scenario-based questions. The responsible and critical use of AI chatbots can enhance medical education, making it an essential aspect to integrate into the educational system.

## Introduction

Artificial intelligence (AI) has been taking significant steps in various fields, including health care and education. The advent of AI chatbots, in particular those built on large language models (LLMs), has opened up new possibilities for enhancing medical education, transforming the way we train future health care professionals. LLMs are a type of generative AI that has been trained on a massively large corpus of textual data from the web, specifically architected to help generate text-based content. AI chatbots have been increasingly used in health care applications, for example, to provide education and support to patients with chronic diseases [1] and to increase COVID-19 vaccine confidence and acceptance [2].

The advent of LLMs has renewed interest toward the potential of AI in the education field, mainly to serve as an assistant for educators and as a virtual tutor for students [3]. For example, CS50, an introductory course in computer science held by Harvard University, plans to use AI to grade assignments, teach coding, and personalize learning tips [4]. Medical education is no exception, with papers exploring examples of AI chatbot applications, including the generation of accurate and versatile clinical vignettes, improving personalized learning experiences, and being an adjunct in group learning [5,6]. For example, the MedQA data set is a well-known data set containing multiple-choice questions collected from real-world professional examinations; it is used as an international benchmark to test the capabilities of AI models in the health care domain [7]. MedQA includes questions from the United States Medical Licensing Exam (USMLE), a set of 3 standardized tests of expert-level knowledge. A recent paper tested ChatGPT performances on the USMLE, showing results near the passing threshold of 60% accuracy. The authors suggested that, based on this result, ChatGPT may potentially support students in the medical education field [8].

The current state-of-the-art model on MedQA performance is Med-PaLM 2, reaching a score of 86.5% in this benchmark [9]. GPT-4 achieves strong performance in many languages, including Italian, on the massive multitask language understanding benchmark, which is a data set of multiple-choice questions not specific to the health care domain [10]. However, the testing of LLMs on medical multiple-choice questions in languages other than English is still limited and worth exploring for its implications on medical education around the world.

This paper aims to evaluate the feasibility of using AI chatbots as educational support tools in public health training in Italy, specifically in the context of vaccination. We compared the performance of different AI chatbot models in answering questions related to vaccination, providing insights into the potential and limitations of AI in medical education.

## Methods

### Study Design

In Italy, since 2015, there has been a national admission test to medical residency after medical school called Prova Nazionale per l’Ammissione dei Medici alle Scuole di Specializzazione di Area Sanitaria*,* hereafter referred to as the Italian National Medical Residency Test (SSM). This test consists of 140 expert-level multiple-choice questions regarding various medical subjects (eg, cardiology and orthopedics), and it is administered in Italian. Each candidate has a specific amount of time (usually 210 minutes) to answer the questionnaire. Based on the scoring on this test, a national ranking is drawn up and each candidate can choose the specific medical residency school they want to enroll in [11].

We chose to focus on vaccination-related questions from the SSM due to their relevance in public health training, their complexity, and their controversial nature in public discourse. The topic of vaccination is related to phenomena of extreme importance, such as infodemics and vaccine hesitancy, which the World Health Organization has identified as one of the top 10 threats to global health [12,13]. We conducted a comparative analysis of different AI chatbots based on LLMs, including Bing Chat, ChatGPT, Chatsonic, Google Bard, and YouChat, in answering a set of questions related to vaccination. These questions were selected from the SSM to ensure their relevance and applicability to the topic of vaccination [14-18]. Our study did not only assess the accuracy of the responses provided by these AI chatbots but also reported a use-case of AI chatbots as assistants for the correction of a test in a real-world scenario of medical education. The completeness of the information, reliability of the sources cited, and use of technical language was discussed between medical students and lecturers with research experience on the use of LLMs in public health. Since good performances by LLMs on medical question answering tasks are necessary but not sufficient to demonstrate their applicability in medical education, we also provided an example of the use of AI chatbots as education support tools for medical students.

### LLM Chatbot Selection

In our study, we chose specific chatbots based on their availability and accessibility. We decided to select only chatbots based on LLMs that use a transformer architecture, as these models can be considered the current gold standard for natural language processing tasks. Our selection was driven by the chatbots’ web-based user interface availability, which obviated the need for model application programming interface use. This methodology enabled us to assess the effectiveness of these chatbots when used by a nontechnical audience, like medical students. Although LLMs fine-tuned for the health care domain, such as Med-Palm2 [9], may demonstrate superior performance in certain contexts, it is pertinent to recognize that their access and use typically necessitate technical expertise via an application programming interface. Consequently, students without a technical background would encounter difficulties in using these resources routinely for academic endeavors.

### Test Extraction and Item Classification

All questions related to vaccination were extracted from the SSM from 2015 (first year of the test) to 2022. The selection process involved a systematic search of questions using targeted keywords related to vaccination (a complete list of the keywords in Italian is provided in Multimedia Appendix 1). The inclusion criteria were as follows: (1) the question must contain any of the keywords and (2) the question must be related to the topic of vaccination. The selection was performed by a single reviewer, and a total of 15 questions were included (Multimedia Appendices 2 and 3).

Furthermore, the questions were classified into the following 3 categories based on their structure [19]:

1. Direct questions: These are straightforward questions that ask for specific information. For example, “What is the composition of the X vaccine?”
2. Scenario-based questions: These questions provide a scenario or case study and then ask a question related to that scenario. They usually require a more comprehensive understanding of a topic, as they often involve applying knowledge to a specific situation. An example from the list is, “A 52-year-old man, with a negative history for COVID-19 and vaccinated with three doses of anti-COVID mRNA vaccine, performs a serological test for anti-SARS-CoV-2 antibodies a month after the third dose. What serological profile do we expect to find?”
3. Negative questions: These questions ask which statements are false or true. They often require a more careful reading, as the use of negation can make them more complex. For instance, “Which of the following statements about vaccine composition is not true?”

### Test Administration to Medical Students and Chatbots

The test was administered using Google Forms [20] to fifth-year medical students as part of their practical training session during the “Hygiene and Public Health” course in April 2023 at the University of Pisa, Italy, before completing all the planned lessons on the topic of vaccination. The form was accessible via a QR code and was anonymous. The test was given to fifth-year medical students because, at the University of Pisa, the public health course is held during the fifth year of medical school. The students were asked to complete the test in 30 minutes.

Subsequently, different AI chatbot models, namely Bing Chat, ChatGPT, Chatsonic, Google Bard, and YouChat, were asked the same set of questions. No prompt was given to the chatbots; the multiple-choice questions were directly copied and pasted into the chat. The responses of the AI chatbots were evaluated on the same scoring basis as the students’ responses, with correct answers scoring 1 point and incorrect or unanswered questions scoring 0 points.

The correction of the test was conducted in the classroom during a dedicated 120-minute session. This involved showing and discussing the solution to the questions provided by one of the chatbots, which was selected based on its performance on the task and its availability. In detail, the criteria for selecting the chatbot for the correction session were as follows: performance above 90% on the task, free web-based availability, and accessible without registration. The main focus of the correction was the critical evaluation of the explanations provided by the chatbot.

Medical students’ feedback was collected anonymously at the end of the training experience through a 3-item questionnaire with a Likert scale (1 to 10) regarding their general satisfaction, willingness to repeat the experience, and ease of use of the tool. In particular, the scale of the 3 items can be translated as follows:

- Item 1: 1=“dissatisfied with the experience,” 10=“very satisfied.”
- Item 2: 1=“I would not repeat the experience,” 10=“I would definitely repeat the experience.”
- Item 3: 1=“the tool is too difficult to be used,” 10=“the tool was very easy to be used.”

Mentimeter [21] was used to collect the feedback right after the correction of the test.

### Statistical Analysis

A Shapiro-Wilk test was conducted to assess the data distribution. In order to investigate any differences in performance between the medical students and AI chatbots, a Mann-Whitney U test was conducted. The rank-biserial correlation was also calculated as a measure of effect size. The performances of the medical students and AI chatbots were compared within each question type, and the Mann-Whitney U test and rank-biserial correlation were calculated for each type of question. All analyses were conducted using Python (Python Software Foundation) with the pandas, matplotlib, seaborn, and scipy libraries. The source data are available in Multimedia Appendix 4 .

### Ethical Considerations

The questionnaire administered in our study was an integral part of the educational activities of the course, serving as a self-assessment tool for the voluntarily participating students. It was designed to maintain the anonymity of the participants and did not collect any personal data. According to the University of Pisa teaching regulations, ethical approval was not necessary for this study as the data were completely anonymous from the beginning and collected by a link to a web platform where respondents could not be identified, and the results of university tests conducted during regular teaching activities are public and open.

## Results

### Test Completion

The test was completed by 36 medical students and 5 different AI chatbot models (Table 1). ChatGPT and Bing Chat were used in different versions. The total score for each participant was calculated out of a maximum of 15 points. The total number of students enrolled in the public health course was 96, of which 36 (37.5%) voluntarily completed the questionnaire.

**Table 1:** The performance of various artificial intelligence (AI) chatbot models in answering the 15 questions selected from the Italian National Medical Residency Test.

AI chatbot	Mode^a^	LLM^b^ model	Score (N=15), n (%)	Not answered	Date of completion
ChatGPT	3.5	GPT3.5	12 (80)	1	April 14, 2023
ChatGPT	4.0	GPT4	15 (100)	—^c^	July 13, 2023
ChatGPT	4.0 plugin Scholar AI	GPT4	15 (100)	—	July 13, 2023
Bing Chat	Precise	—	15 (100)	—	April 13, 2023
Bing Chat	Creative	—	14 (93)	—	April 12, 2023
Bing Chat	Balanced	—	11 (73)	—	April 13, 2023
Google Bard	—	LaMDA^d^	7 (47)	2	July 13, 2023
YouChat	—	—	10	2	April 14, 2023
Chatsonic	—	GPT4	11	1	April 14, 2023

^a^The specific mode or version of the AI chatbot used.

^b^LLM: large language model.

^c^Not applicable.

^d^LaMDA: Language Model for Dialogue Applications

Shapiro-Wilk tests indicated normal distributions for the total scores of both chatbots and students but nonnormal distributions for all the subcategories of questions (direct, scenario-based, and negative) for both chatbots and students. For this reason, the Mann-Whitney U was chosen as the statistical test for all the comparisons.

**Figure 1:**
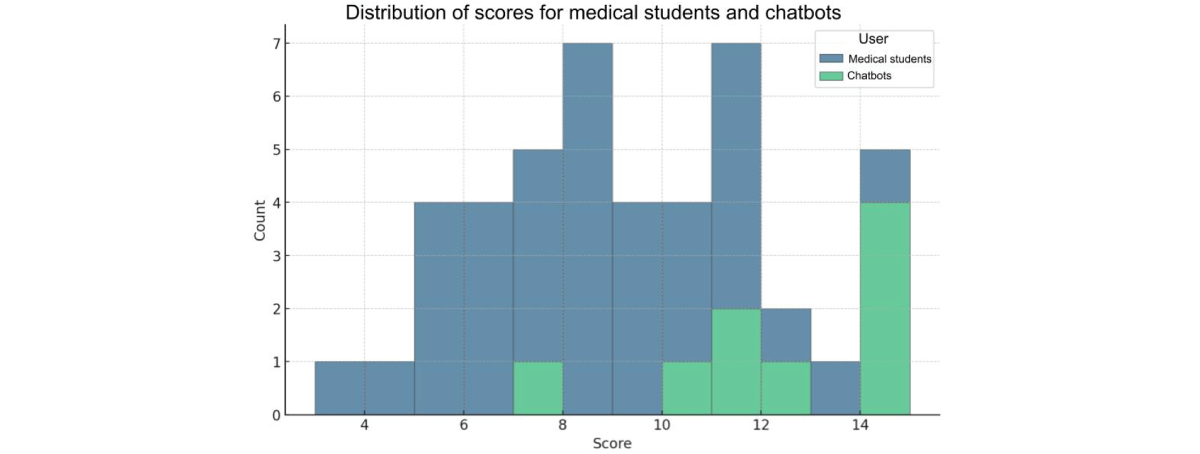
This histogram represents the distribution of overall scores obtained by medical students (in blue) and AI chatbots (in green) on the vaccine-related Italian National Medical Residency Test questions. Each bar represents the stacked number of students or chatbots that achieved a particular score. The scores are represented on the x-axis, and the number of students or chatbots achieving each score is represented on the y-axis.

**Figure 2:**
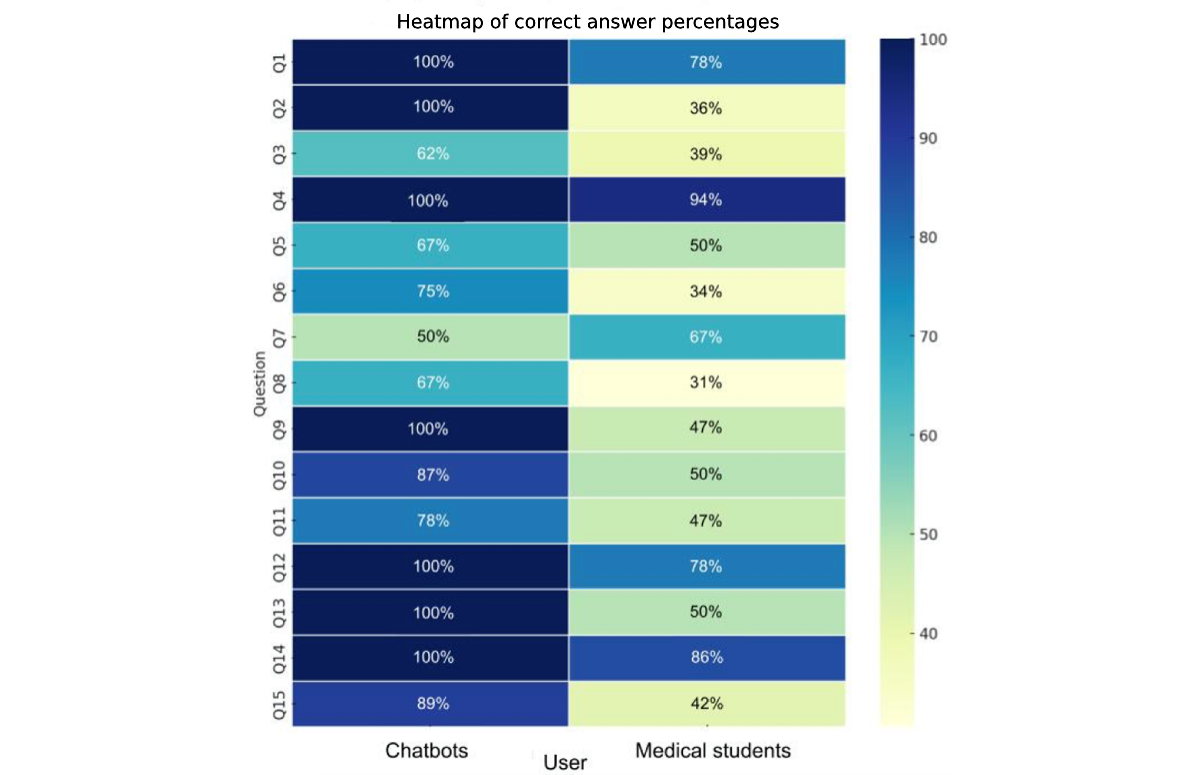
This heat map visualizes the percentage of correct answers provided by medical students and AI chatbot models for each question on the vaccine-related test. The questions are represented on the y-axis, and the user types (medical students or AI chatbot models) are represented on the x-axis. The color intensity in each cell corresponds to the percentage of correct answers, with darker shades representing higher percentages. The percentages are also annotated within the cells for easier reference.

On average, out of 15, medical students scored 8.22 (SD 2.65; median 8, IQR 4-12; range 3-15), while the AI chatbot models scored higher, with an average score of 12.22 (SD 2.77; median 12, IQR 8-15; range 7-15). The distribution of scores is displayed in Figure 1. Details regarding the accuracy of chatbots and medical students on each single question are provided in Figure 2.

### Performances on the Test: Comparison Between Medical Students and AI Chatbots

A Mann-Whitney U test was conducted to compare the total scores of the medical students and AI chatbots. The result indicated a statistically significant difference (U=49.5, *P*<.001). The rank-biserial correlation (r=0.69) suggested a large effect size, indicating a meaningful difference between the performances of the 2 groups.

**Figure 3:**
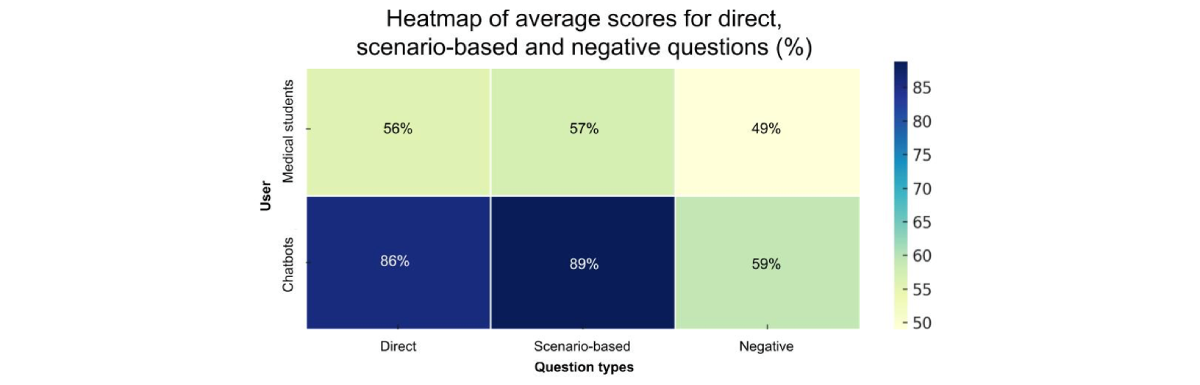
This heat map displays the percentages of correct answers for the 2 groups of chatbots and medical students in each specific category of questions. The color intensity in each cell corresponds to the percentage of correct answers, with darker shades representing higher percentages. The percentages are also annotated within the cells for easier reference.

The 15 items of the test were classified as following: 7 direct questions, 5 scenario-based questions, and 3 negative questions. Details of the classification can be found in Multimedia Appendix 2. Chatbots scored an average of 6.00 (SD 1.12; median 6, IQR 4-7) out of 7 on direct questions, 4.44 (SD 0.73; median 5, IQR 4-5) out of 5 on scenario-based questions, and 1.78 (SD 0.53; median 2, IQR 0-3) out of 3 on negative questions. Students scored an average of 3.89 (SD 1.14; median 4, IQR 2-6) out of 7 on direct questions, 2.86 (SD 1.31; median 3, IQR 1-5) out of 5 on scenario-based questions, and 1.47 (SD 1.00; median 1, IQR 0-2) out of 3 on negative questions. The percentage of correct answers to each type of question for both groups can be found in Figure 3.

For direct questions, the Mann-Whitney U test showed a statistically significant difference in the scores of medical students and AI chatbots (U=33.5, *P*<.001). The rank-biserial correlation was 0.79, indicating a large effect size. For scenario-based questions, the Mann-Whitney U test also showed a statistically significant difference in scores (U=52.5, *P*=.002). The rank-biserial correlation was 0.68, suggesting a large effect size. However, for negative questions, there was no statistically significant difference in scores (U=137.5, *P*=.48). The rank-biserial correlation was 0.151, indicating a small effect size.

### Report of the Educational Experience

Bing Chat (creative mode) was chosen to conduct the corrections in the classroom due to its good performance on the task (score: 14/15) and its availability at the time of our study.

Throughout the correction process, students actively participated in discussions, critically evaluating the explanations provided by the chatbot. Feedback collected postsession via Mentimeter revealed a high level of satisfaction and ease of use, with scores of 7.9 and 8.2, respectively, on a 10-point Likert scale. The students commended the novel and interactive format, stating that it added a fresh dimension to the traditional teaching approach, and showed a strong desire to repeat the experience (7.6/10).

## Discussion

### Principal Findings

Our paper explored the role of AI chatbots, particularly those constructed upon LLMs (ie, ChatGPT), in medical education and their potential to support learning and training in public health through a practical use-case experience. The results of our feasibility study showed that LLM-based chatbots can correctly answer complex health-related multiple-choice questions in Italian in the specific domain of vaccination, proving to possibly be a supporting educational tool in this specific setting. By using questions from the SSM, we not only evaluated the accuracy of the chatbots’ responses but also examined a real-world application of AI in providing an explained correction of a medical admission test. Bing Chat (creative mode) was chosen for the correction in class because, while it was not the best-performing chatbot, it provided longer and more in-depth answers to each question, thus providing a better ground for classroom discussion with students. ChatGPT was temporarily unavailable in Italy following an action of the President of the Italian Data Protection Authority for breaches of the European legislation on personal data processing and protection by OpenAI [22].

The chatbots analyzed exhibited high-level performance that was, on average, higher than the performance of the medical students. The chatbots showed a statistically significant superiority for direct and scenario-based questions, while they were less accurate on negative questions (not statistically significant). The performances of chatbots on this specific task relied on various factors and could be further improved by using prompt engineering and techniques such as chain-of-thought prompting [23].

Notably, good performance alone is not enough for the useful and safe adoption of these tools in real-world applications for medical education purposes. Especially in the medical domain, it is better to promote awareness of the benefits and limits of LLMs rather than prohibiting students from using them [24]. The critical evaluation of the answers provided by the chatbot not only enhanced students’ understanding of the correct responses but also stimulated conversations about the underlying concepts, resulting in a positive attitude of the participating students toward the tool [25]. In fact, the students reported a general satisfaction and a willingness to repeat the educational experience proposed in our study.

As suggested later by Cooper and Rodman [26], as medical educators, we took an activist approach trying to integrate AI into physician training, with the objective of preparing our students for safe and appropriate use of this technology in health care. In the current educational landscape, while the potential of LLMs as teaching tools is evident, their incorporation into traditional pedagogical methods demands planning. LLMs can be a useful support tool within different phases of teaching. In the introduction of new topics, they can act as supplementary informational sources, helping students to grasp foundational concepts quickly [5,27]. During in-depth discussions or tutorials, LLMs can serve as interactive tools to challenge students’ understanding, offering real-time feedback [4]. Moreover, in the revision phase, these models can be pivotal in addressing specific queries, clarifying doubts, and reinforcing knowledge through simulated question and answer sessions.

Our approach allows group discussions stimulating critical thinking about the potentiality and limits of AI chatbots in medical education. In fact, it is crucial to introduce students to the limitations of LLMs, such as their reliance on biased data, limited up-to-date knowledge, variable performances over time, and the potential for generating incorrect or false information [3,27]. The issue of “hallucinations'' is particularly concerning in medical education and has to be properly discussed with students due to the possible fabrication of scientific references among other false or misleading information [28,29]. Even if the reliability of scientific references cited by ChatGPT and other LLMs is rapidly increasing thanks to their ability to browse the web and the use of plug-ins, such as ScholarAI, that seamlessly integrate peer-reviewed article searches into ChatGPT conversations, the need to demand a deep and critical check of the sources cited by LLMs and treat them as guilty until proven innocent remains [30].

A critical use of this tool should also be encouraged to counter the deskilling derived from an overreliance on it—students might eventually lose their abilities to produce original ideas and present proper arguments to prove their statements. Furthermore, chatbots cannot be used as substitutes in clinical reasoning, and specific training, through case studies and simulations, should be foreseen in medical school [31]. Since students, residents, and fellows are already using such tools, it is our duty to guide the academic community in raising awareness rather than prohibiting, or worse ignoring, the change.

### Limitations

Our study has some limitations. The sample size of medical students was relatively small, which may limit the generalizability of the results. A larger sample size could offer a more comprehensive and reliable reflection of the performance of medical students. Moreover, all the questions used for the test focused solely on the topic of vaccination. While this focus provided valuable insights into the performance of the AI chatbots and students in this specific area, it may not fully represent their proficiency across a wider array of medical topics. Additionally, due to restrictions on time and availability, only one chatbot was thoroughly used and evaluated with the students in class. As highlighted in a recent paper [32], the performance of ChatGPT on different tasks seems to substantially change over time, at times worsening. Even if this behavior has not been demonstrated for medical questions yet, it could potentially reduce the long-term reliability of our results.

### Future Perspectives

The present study offers insights into the potential role of AI chatbots as support tools in training. There are multiple stages in the individual training pathway where students can benefit from the support of this technology. The cited Harvard example [4] is just one of many potential applications. In the medical field, AI-powered chatbots can assist students in conducting targeted searches for scientific literature, helping them find relevant and reliable references for their studies. Essentially, the chatbots could serve as an interface that may guide students to the best available learning resources, discarding irrelevant or less useful materials. This approach could offer personalized training, catering to individual interests and personal learning needs. However, it should not only focus on knowledge components, potentially neglecting the development of competencies, as defined by the World Health Organization [33]. The implementation of mutable virtual simulation scenarios could address the implementation of specific skills and attitudes; in this use case, students could face a simulation that was not based on predetermined algorithmic scripts but rather on a virtual interlocutor with a variable and human-like approach powered by AI. In this way, it may be possible to develop an experiential approach similar to a specific real-world scenario (eg, an interview of parents on vaccine adverse effects), which would be useful for training students’ communication and practical skills in public health.

### Future Studies

In the future, we aim to investigate the performance of chatbots across all questions from the SSM to assess how well the AI models can navigate a broader and more diverse range of medical subjects. Such an analysis would allow us to deeply evaluate the ability of chatbots to comprehend and respond accurately in Italian, evaluating linguistic proficiency gaps that might need to be addressed in future model development for the tools to be actually used by Italian medical students. Another aspect of this future study would be a comparison between the AI chatbots’ performance and the actual results obtained by Italian doctors, providing a significantly wider benchmark for the Italian language.

Further studies are needed to assess if the integration of AI tools in public health medical training may improve the acquisition of knowledge and performances in final exams. In this way, we think that starting with a practical example of the application of a chatbot based on LLMs can be a beginning for experimenting with AI in support of training for health professionals, with the prospect of expanding this range of application to orient us toward the innovations in training proposed by supranational and national organizations.

Our feasibility study provided a real-world example of the application of AI tools in support of training for health professionals in public health. It demonstrated a good reliability of the tools used and a high satisfaction of the students for this type of practical activity, supporting the possible use of AI for medical education in public health. Further studies should be encouraged to explore other possible applications of AI-based tools in health care training in order to assess if they improve the performance of the students and to guide their awareness and critical use.

## Appendix

Multimedia Appendix 1 Keywords used for the filtering of questions from the SSM to identify vaccine-related questions.

Multimedia Appendix 2 List of questions used in the study. This table presents the 15 questions selected through keywords from the Italian National Medical Residency Test (SSM) translated into English. Each question is categorized by type: direct, scenario-based, or negative. The questions cover various topics related to vaccination and are used to evaluate the performance of AI chatbots in providing accurate and comprehensive responses.

Multimedia Appendix 3 Original questions in Italian extracted from Italian National Medical Residency Tests (SSMs) from 2015 to 2022.

Multimedia Appendix 4 Raw data.

## Abbreviations

AI: artificial intelligence
LLM: large language model
SSM: Italian National Medical Residency Test
USMLE: United States Medical Licensing Exam

## Data Availability

All raw data are available in Multimedia Appendix 4.
