# Supplementary material for: Exploring the Possible Use of AI Chatbots in Public Health Education: Feasibility Study
Source: JMIR Med Educ. 2023 Nov 1;9:e51421. doi: 10.2196/51421 (PMC10652189; doi:10.2196/51421)
Supplement: Multimedia Appendix 1 [file mededu_v9i1e51421_app1.docx]

## Supplementary file

Keywords utilized for the filtering of questions from SSM to identify vaccine related questions:

vacc*

immunizzazion*

inoculazion*

immunità

prevenzione

morbillo

poliomielite
IPV

OPV

COVID-19

SARS-CoV-2

orecchioni

rosolia

varicella

MPR

MPR-V

Haemophilus influenzae tipo B

HiB

meningococco

MEN

pneumococco

tubercolosi

epatite
HAV
HBV

pertosse

difterite

tetano

dTpa

esavalente

tetravalente

papillomavirus umano

HPV

influenza

FLU

antigene

anticorpo

calendar*
