# Supplementary material for: Exploring the Possible Use of AI Chatbots in Public Health Education: Feasibility Study
Source: JMIR Med Educ. 2023 Nov 1;9:e51421. doi: 10.2196/51421 (PMC10652189; doi:10.2196/51421)
Supplement: Multimedia Appendix 2 [file mededu_v9i1e51421_app2.docx]

**Table 1S:** **List of Questions Used in the Study**. This table presents the 15 questions selected through keywords from the Italian National Medical Residency Test (SSM) translated in English. Each question is categorized by type: 'Direct', 'Scenario-Based', or 'Negative'. The questions cover various topics related to vaccination and are used to evaluate the performance of AI chatbots in providing accurate and comprehensive responses.

| **Question** | **English Translation** | **Type** |
| --- | --- | --- |
| Q1 | A 52-year-old man, with no medical history of COVID-19 and vaccinated with three doses of mRNA anti-COVID vaccine, undergoes a serological test for anti-SARS-CoV-2 antibodies one month after the third dose. What serological profile do we expect to find?  A: Positivity for IgG anti-Spike protein and negativity for IgG anti-N protein  B: Negativity for IgG anti-Spike protein and positivity for IgG anti-N protein  C: Positivity for IgG anti-Spike protein and for IgG anti-N protein  D: Negativity for IgG anti-Spike protein and for IgG anti-E protein  E: Positivity for IgG anti-E protein | scenario-based |
| Q2 | In the case of diseases preventable by vaccination, the eradication goal is achieved when:  A: Globally, the incidence of the disease is consistently 0, and the causative agent is no longer present in nature  B: Globally, the incidence is consistently 0, even in the presence of infected animal or environmental reservoirs  C: In one or more WHO regions, the incidence of the disease is consistently 0, even in the presence of infected animal or environmental reservoirs  D: In one or more regions, the definitive eradication of animal reservoirs is documented, regardless of the number of cases still present  E: Globally, the vaccination coverage is > 90% | direct |
| Q3 | The anti-SARS-CoV-2 vaccines, both mRNA and viral vector, used for the vaccination campaign in Italy:  A: Aim to stimulate an immune response against the Spike protein that is synthesized in vivo  B: Aim to stimulate an immune response against the Spike protein contained in the vaccine preparation and synthesized in the laboratory  C: Provide more effective protection against infection than the disease  D: Are polyvalent as they are prepared from different variants of the virus, including the omicron variant  E: Should never be administered to women who are pregnant (confirmed or suspected) or breastfeeding | direct |
| Q4 | For which of the following sites of carcinoma is there currently a vaccine against the main etiological agent, capable of preventing over 90% of cases if administered before the onset of sexual activity?  A: Cervix  B: Ovary  C: Endometrium  D: Breast  E: Fallopian Tubes | direct |
| Q5 | Indicate which of the following statements about the composition of vaccines is true:  A: Anti-measles-mumps-rubella-varicella is made up of live attenuated viruses  B: Anti-polio IPV is made up of live attenuated viruses  C: Anti-pertussis is made up of conjugated capsular antigens  D: Anti-hepatitis A is made up of surface antigens of the virus  E: Anti-meningococcal B is made up of conjugated capsular antigens | negative |
| Q6 | Gaia's mother, alarmed by media misinformation on the subject of vaccines and the adverse reactions that occurred in her two-year-old grandson, turns to the family pediatrician to ask for information and advice. The pediatrician reassures her about the safety and efficacy of all vaccines and specifies that:  A: The pneumococcal vaccination is recommended  B: The rotavirus vaccination has become mandatory  C: The meningococcal C vaccination is mandatory  D: The tetravalent vaccination against measles-mumps-rubella-varicella is recommended  E: The vaccination against diphtheria-tetanus-pertussis is no longer mandatory | scenario-based |
| Q7 | Post-exposure tetanus prophylaxis may require the use of the tetanus vaccine, possibly combined with the administration of immunoglobulins. In an adult subject, in which case should neither active nor passive prophylaxis be performed?  A: Only if the subject has received 3 or more doses of the vaccine no more than 5 years ago  B: Only if the wound is neither severe nor deep, regardless of the subject's vaccination status  C: If they have received a vaccination cycle, even incomplete, in the last 10 years  D: If the subject has been fully vaccinated during childhood  E: In the case of a previous episode of tetanus in a subject who has never been vaccinated | negative |
| Q8 | Which composition of the following vaccines available today is correctly indicated?  A: The measles vaccine is made up of attenuated viruses  B: The anti-tuberculosis vaccine is made up of extracted and purified antigens  C: The Haemophilus influenzae type B vaccine is made up of inactivated viruses  D: The serogroup B meningococcal vaccine is made up of capsular polysaccharide antigens  E: The pneumococcal vaccine is a polyvalent vaccine made up of outer membrane proteins | negative |
| Q9 | A 3-year-old child presents with fever and maculopapular rash, initially appearing in the retroauricular region and on the forehead, and then spreading in a head-to-toe direction to the whole body. You diagnose measles. What therapy do you recommend?  A: Antipyretics  B: Acyclovir  C: Immediate administration of the vaccine with live attenuated virus  D: Amoxicillin/clavulanic acid | scenario-based |
| Q10 | In Italy, what type of polio vaccine is currently being used?  A: Vaccine with killed microorganisms  B: Vaccine with live attenuated microorganisms  C: Vaccine with fragmented microorganisms  D: Subunit antigen vaccine | direct |
| Q11 | Live attenuated vaccines:  A: can be inactivated by circulating antibodies (including maternal ones)  B: can be administered to subjects with immunosuppression, although they are not always proven effective  C: involve a response that is predominantly antibody-based and not cellular  D: generally require 5 doses (and boosters) | direct |
| Q12 | Poliomyelitis is a viral infectious disease sometimes characterized by the appearance of paralysis in the limbs. Since 1988, the WHO has established a global program for the eradication of the disease, and the European region was declared polio-free in 2002. What are the best preventive measures to adopt to maintain the polio-free status?  A: Vaccination and Surveillance of Acute Flaccid Paralysis  B: Isolation and adoption of contumacy rules  C: Disinfection, sterilization, and pest control  D: Notification and prompt administration of antiviral drugs | scenario-based |
| Q13 | Rubella is a contagious viral disease that becomes dangerous if contracted during pregnancy because it can cause spontaneous abortion, intrauterine death, or severe fetal malformations. The National Plan for the Elimination of Measles and Rubella (PNEMoRc) 2010-2015 included among its objectives the reduction of the incidence of congenital rubella to less than 1 case per 100,000. Rubella prevention can be done through:  A: vaccine with attenuated virus  B: vaccine with inactivated virus  C: subunit vaccine  D: recombinant DNA vaccine | scenario-based |
| Q14 | Tetanus is an infectious disease caused by a potent toxin produced by the bacterium Clostridium tetani. The prevention of the disease is based on vaccination, the correct administration schedule of which is:  A: 1st dose at time zero, 2nd dose after 4-6 weeks, 3rd dose after 6-12 months. Booster every 10 years  B: 1st dose at time zero, 2nd dose after 4-6 weeks. Booster after 2 years  C: 1st dose at time zero, 2nd dose after 5 years, 3rd dose after 10 years  D: 1st dose at time zero, 2nd dose after 4-6 weeks, 3rd dose after 6 years | direct |
| Q15 | What does the vaccination schedule for the hepatitis A virus in adults provide?  A: An initial dose and a booster at 6-18 months  B: An initial dose, a second dose at 1 month  C: An initial dose, a second dose at 1 month, and a third dose at 6 months  D: No vaccine exists | direct |

## 
