# Supplementary material for: Exploring the Possible Use of AI Chatbots in Public Health Education: Feasibility Study
Source: JMIR Med Educ. 2023 Nov 1;9:e51421. doi: 10.2196/51421 (PMC10652189; doi:10.2196/51421)
Supplement: Multimedia Appendix 3 [file mededu_v9i1e51421_app3.docx]

## Supplementary file

Original questions extracted by the SSM2015, SSM2016, SSM2017, SSM2018, SSM2019, SSM2020, SSM2021 and SSM2022

(A is always the correct answer)

Q1 Scenario-based

Domanda 108: (codice domanda: ssm2022248)

Un uomo di 52 anni, con negatività anamnestica per COVID-19 e vaccinato con tre dosi di vaccino mRNA anti-COVID, esegue un test sierologico per anticorpi anti SARS-CoV-2 a distanza di un mese dalla terza dose. Quale profilo sierologico ci aspettiamo di trovare?

A: Positività per IgG anti-proteina Spike e negatività per IgG anti-proteina N

B: Negatività per IgG anti-proteina Spike e positività per IgG anti-proteina N

C: Positività per IgG anti-proteina Spike e per IgG anti-proteina N

D: Negatività per IgG anti-proteina Spike e per IgG anti-proteina E

E: Positività per IgG anti-proteina E

Q2 Direct

Domanda 133: (codice domanda: ssm2022273)

Nel caso di malattie prevenibili con la vaccinazione l’obiettivo eradicazione si ottiene quando:

A: A livello globale l’incidenza della malattia è stabilmente pari a 0 e l’agente causale non è più presente in natura

B: A livello globale l’incidenza è stabilmente pari a 0, pur in presenza di serbatoi animali o ambientali infetti

C: In una o più regioni WHO l’incidenza della malattia è stabilmente pari a 0, pur in presenza di serbatoi animali o ambientali infetti

D: A livello di una o più regioni è documentata la definitiva bonifica dei serbatoi animali a prescindere dal numero di casi ancora presenti

E: A livello globale la copertura vaccinale è > 90%

Q3 Direct

Domanda 135: (codice domanda: ssm2022275)

I vaccini anti SARS-CoV-2, a mRNA e a vettore virale, utilizzati per la campagna di vaccinazione in Italia:

A: Mirano a stimolare una risposta immunitaria nei confronti della proteina Spike che viene sintetizzata in vivo

B: Mirano a stimolare una risposta immunitaria nei confronti della proteina Spike contenuta nel preparato vaccinale e sintetizzata in laboratorio

C: Conferiscono protezione più efficace nei confronti dell’infezione che della malattia

D: Sono polivalenti in quanto allestiti a partire da diverse varianti del virus, compresa la variante omicron

E: Non devono mai essere somministrati alle donne in gravidanza (accertata o presunta) o in allattamento

Q4 Direct

Domanda 81: (codice domanda: ssm202110232854)

Per quale delle seguenti sedi di carcinoma esiste attualmente un vaccino contro l'agente eziologico principale, in grado di prevenire oltre il 90% dei casi, se effettuato prima dell'inizio dell'attività sessuale?

A: Cervice

B: Ovaio

C: Endometrio

D: Mammella

E: Salpingi

Q5 Negative

Domanda 135: (codice domanda: ssm202110235554)

Indicare quale delle seguenti affermazioni sulla composizione dei vaccini è vera:

A: Anti-morbillo-rosolia-parotite-varicella è costituito da virus vivi attenuati

B: Anti-poliomielite IPV è costituito da virus vivi attenuati

C: Anti-pertosse è costituito da antigeni capsulari coniugati

D: Anti-epatite A è costituito da antigeni di superficie del virus

E: Anti-meningococco B è costituito da antigeni capsulari coniugati

Q6 Scenario-based

Domanda 133: (codice domanda: ssm20203818454)

La mamma di Gaia, allarmata dalla disinformazione mediatica sulla questione vaccini e dalle reazioni avverse occorse al nipotino di due anni, si rivolge al pediatra di famiglia per chiedere informazioni e consiglio. Il pediatra la rassicura sulla sicurezza ed efficacia di tutti i vaccini e le precisa che:

A: La vaccinazione anti pneumococco è raccomandata

B: La vaccinazione anti rotavirus è diventata obbligatoria

C: La vaccinazione anti meningococco C è obbligatoria

D: La vaccinazione tetravalente anti morbillo-rosolia-parotite-varicella è raccomandata

E: La vaccinazione anti difterite-tetano-pertosse non è più obbligatoria

Q7 Negative

Domanda 134: (codice domanda: ssm20203818504)

La profilassi antitetanica post-esposizione può richiedere l'utilizzo del vaccino antitetanico eventualmente associato alla somministrazione di immunoglobuline. In un soggetto adulto in quale caso NON deve essere effettuata né la profilassi attiva né quella passiva?

A: Solo se il soggetto ha ricevuto 3 o più dosi di vaccino da non più di 5 anni

B: Solo se la ferita non è grave né profonda a prescindere dallo stato vaccinale del soggetto C: Se ha ricevuto un ciclo vaccinale anche incompleto negli ultimi 10 anni

D: Se il soggetto è stato completamente vaccinato durante l'infanzia

E: In caso di pregresso episodio di tetano in soggetto mai vaccinato

Q8 Negative

Domanda 55: (codice domanda: ssm2019107) :

Quale composizione dei seguenti vaccini oggi disponibili è correttamente indicata?

A: Il vaccino anti-morbillo è costituito da virus attenuati

B: Il vaccino anti-tubercolare è costituito da antigeni estratti e purificati

C: Il vaccino anti-Haemophilus influenzae tipo B è costituito da virus inattivati

D: Il vaccino anti-meningococco di sierogruppo B è costituito da antigeni polisaccaridici capsulari

E: Il vaccino anti-pneumococco è un vaccino polivalente costituito da proteine della membrana esterna

Q9 Scenario-Based

Scenario 9:

Un bimbo di 3 anni presenta febbre ed esantema maculopapuloso comparso inizialmente in regione retroauricolare e alla fronte e quindi diffusosi in senso craniocaudale a tutto il corpo. Ponete diagnosi di morbillo. Domanda #20 (codice domanda: n.110) - (riferita allo scenario n.9) : Quale terapia consigliate?

A: Antipiretici

B: Aciclovir

C: Somministrazione immediata del vaccino con virus vivo attenuato

D: Amoxicillina/acido clavulanico

Q10 Direct

Domanda #6 (codice domanda: n.696) : In Italia che tipo di vaccino antipolio viene utilizzato attualmente?

A: Vaccino a microrganismi uccisi

B: Vaccino a microrganismi vivi attenuati

C: Vaccino a microrganismi frammentati

D: Vaccino a subunità antigenica

Q11 Direct

Domanda #4 (codice domanda: n.694) : I vaccini vivi attenuati:

A: possono essere inattivati da anticorpi circolanti (inclusi quelli materni)

B: possono essere somministrati a soggetti con immunosoppressione, anche se non sempre si dimostrano efficaci

C: prevedono una risposta prevalentemente anticorpale e non cellulare

D: generalmente richiedono 5 dosi (e boosters)

Q12 Scenario-based

Scenario 1: La poliomielite è una malattia infettiva virale talora caratterizzata dalla comparsa di paralisi agli arti. A partire dal 1988 l'OMS ha istituito un programma mondiale per l'eradicazione della malattia e la regione europea è stata dichiarata polio-free nel 2002. Domanda #9 (codice domanda: n.699) - (riferita allo scenario n.1) : Quali sono le migliori misure preventive da adottare per mantenere lo stato di polio-free?

A: Vaccinazione e Sorveglianza della Paralisi Flaccida Acuta

B: Isolamento e adozione di norme contumaciali

C: Disinfezione, sterilizzazione e disinfestazione

D: Notifica e pronta somministrazione di farmaci antivirali

Q13 Scenario-based

Scenario 2: La rosolia è una malattia virale contagiosa che diventa pericolosa se contratta durante la gravidanza perché può provocare aborto spontaneo, morte intrauterina o gravi malformazioni fetali. Il Piano nazionale di eliminazione del morbillo e della rosolia (PNEMoRc) 2010-2015 includeva tra i suoi obiettivi la riduzione dell'incidenza della rosolia congenita a meno di 1 caso su 100.000. Domanda #10 (codice domanda: n.700) - (riferita allo scenario n.2) : La prevenzione della rosolia può essere effettuata mediante:

A: vaccino a virus attenuato

B: vaccino a virus inattivato

C: vaccino a subunità

D: vaccino a DNA ricombinante

Q14 Direct

Domanda #3 (codice domanda: n.793) :

Il tetano è una malattia infettiva causata da una potente tossina prodotta dal batterio Clostridium tetani. La prevenzione della malattia si basa sulla vaccinazione, il cui corretto schema di somministrazione è:

A: 1° dose al tempo zero, 2° dose dopo 4-6 settimane, 3° dose dopo 6-12 mesi. Richiamo ogni 10 anni

B: 1° dose al tempo zero, 2° dose dopo 4-6 settimane. Richiamo dopo 2 anni

C: 1° dose al tempo zero, 2° dose dopo 5 anni, 3° dose dopo 10 anni

D: 1° dose al tempo zero, 2° dose dopo 4-6 settimane, 3° dose dopo 6 anni

Q15 Direct

Domanda #17 (codice domanda: n.107) : Cosa prevede la scheda vaccinale per il virus dell'epatite A nell'adulto?

A: Una dose iniziale e un richiamo a 6-18 mesi

B: Una dose iniziale, una seconda dose a 1 mese

C: Una dose iniziale, una seconda dose a 1 mese e una terza dose a 6 mesi

D: Non esiste vaccino

## 
